# Esmarch’s Method

**Published:** 1875-01

**Authors:** 


Esmarch's Method.—In the *Medical Times*, Prof. Sands says: Although he had not the means of making an exact comparison, he believed that the percentage of recoveries in these primary amputations—which were nearly all performed upon hospital patients—is somewhat larger than is commonly observed in such operations.

Of sixteen cases of excision of the joints, only one proved fatal, the cause of death being pyæmia; another case will probably end fatally, however, as when last heard from the patient was reported to have tetanus. Finally, the list includes two more deaths: one following an operation for necrosis—the only fatal case out of thirty-six—and the other an operation for ligation of the ulnar artery, performed for a wound near the wrist.

Upon the whole, the method is one concerning which he spoke in the highest terms. It is one of the most valuable surgical expedients of modern times, and needs only proper knowledge and skill in its application.

Prof. James R. Wood, M.D., remarked that he had operated twenty-five times, each time employing this method, without bad results, with the exception of one case, mentioned in the table of Dr. Sands. He has repeatedly operated, both in hospital and private practice, in young and old, men and women, and has had no trouble whatever, and he has "fallen in love with the operation." The surgeon who formerly performed his operations by groping in the darkness can now witness each step of the operation with the eye. "Is there not," said he, "something beautiful in this—something worthy the attention of surgeons?" He was not in favor of the cord, but of the bandage.

Prof. Frank H. Hamilton has made eight operations, employing the bloodless method—four amputations and four exsections—and he is highly pleased with the success attained. He has had no accident that he can possibly attribute to Esmarch's method. He did not look upon the cord with favor. He preferred a bandage.

Prof. Markoe was satisfied with the efficacy of the method in preventing hemorrhage. He has had no accidents from this cause. He mentioned a case where the cord had been used. There was no sloughing at the time, but five or six weeks after the operation, just at the point where the cord had made pressure, a sloughing occurred down or nearly down to the bone. The case, however, recovered. With this single exception, he had experienced no bad results.

Dr. Krackowizer, who was the first to employ this method in this city, on the 22d of October, 1873, is still in favor of it. Instead of the rope or elastic webbing or bandage, which is objectionable on account of the possibility of its accumulating filth, and because of the inability of the surgeon always to secure the most approved articles, he thought it well to extemporize a bandage whenever practicable. He had from the first objected to the cord, and was in the habit of splitting a common india rubber hose, an inch or so in diameter. This served the principle and the purpose.

Dr. Watson spoke of a case occurring in the town of Niagara, New York. The wrist was crushed. Operation was performed at the lower third of the fore-arm, by Esmarch's method. On the second day after the operation, the tissues became somewhat discolored. On the fourth day, the limb in the neighborhood of the stump became gangrenous. The unfavorable character of the case rendered a second operation necessary. He has not heard further from the case.

Dr. Parker had not had any personal experience in the matter, but suggested, in a general way, various untoward results which might possibly happen.

To sum up, the sentiment of the meeting appeared to be largely in favor of this method. It was looked upon as second in importance, as relates to the whole field of surgery, to the discovery of anæsthesia; and, like the subject of anæsthetics, it requires close and extended study to bring it to a state of perfection.

